# Comparative analyses of bidirectional promoters in vertebrates

**DOI:** 10.1186/1471-2105-9-S6-S9

**Published:** 2008-05-28

**Authors:** Mary Qu Yang, James Taylor, Laura Elnitski

**Affiliations:** 1 Genome Technology Branch, NHGRI, NIH, MD, USA; 2 Courant Institute of Mathematical Sciences, New York University, NY, USA

## Abstract

**Background:**

Orthologous genes with deep phylogenetic histories are likely to retain similar regulatory features. In this report we utilize orthology assignments for pairs of genes co-regulated by bidirectional promoters to map the ancestral history of the promoter regions.

**Results:**

Our mapping of bidirectional promoters from humans to fish shows that many such promoters emerged after the divergence of chickens and fish. Furthermore, annotations of promoters in deep phylogenies enable detection of missing data or assembly problems present in higher vertebrates. The functional importance of bidirectional promoters is indicated by selective pressure to maintain the arrangement of genes regulated by the promoter over long evolutionary time spans. Characteristics unique to bidirectional promoters are further elucidated using a technique for unsupervised classification, known as ESPERR.

**Conclusion:**

Results of these analyses will aid in our understanding of the evolution of bidirectional promoters, including whether the regulation of two genes evolved as a consequence of their proximity or if function dictated their co-regulation.

## Background

Bidirectional promoters are defined as the regulatory regions that are shared between two genes, when those two genes are transcribed away from one another [1]. The genes are said to be in a head-to-head arrangement, with their Transcription Start Sites (TSSs) positioned nearby one another. By definition, the intergenic distance between these genes (i.e. the promoter length) can be no greater than 1000 bp [1]. This distance is measured from the TSS of the gene on the left of the promoter to the TSS of the gene on the right of the promoter. Head-to-head genes are spaced at this distance more frequently than expected in the human genome [2], suggesting a regulatory theme in gene expression. We recently showed that the human genome contains more bidirectional promoters than previously recognized [3,4]. Here we map the orthologous regions of bidirectional promoters in seven additional species.

Using the "chains and nets" data from the UCSC Human Genome Browser and the Liftover tool [5], we are able to use the identity of genes on each side of a bidirectional promoter to find the corresponding functional location in other species. The use of these orthologous genes, which were present in the last common ancestor to the species being compared, is important because bidirectional promoters themselves often do not show a strong signal for conserved sequences. This fact makes the assignment of ancestral relationships difficult in these regulatory regions. Because genes flank both sides of bidirectional promoters, they provide markers of the ancestral history. The presence of the same head-to-head genes over long evolutionary time spans facilitates the assignment of orthology at their intervening promoter regions. Our approach complements and extends the work of Li et al. 2006 [6] who examined bidirectional promoters in multiple species, because we are able to explore the ancestral history of bidirectional promoters in eight species simultaneously using whole genome orthology information. Our method uses orthologous genes as anchors that can be traced across vertebrate genomes. We use this information to track the appearance of bidirectional promoters in vertebrate evolution, predict when gene annotations are missing in higher vertebrates and determine which gene functions regulated by these promoters are the oldest.

## Results and discussion

### Orthologous gene pairs identify ancestral patterns of gene regulation

**Figure 1:**
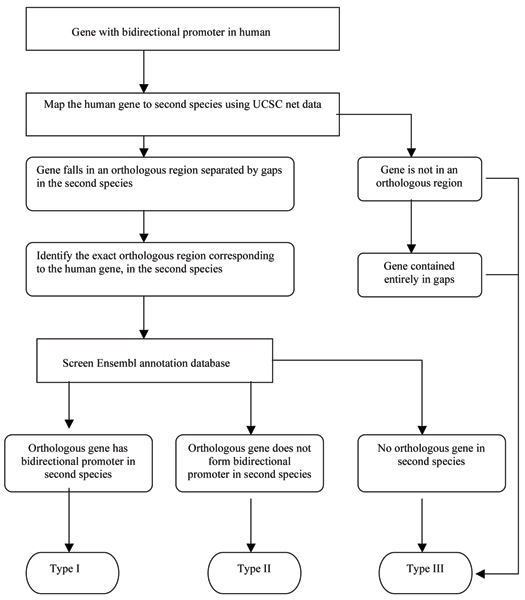
**Flow diagram for mapping orthologous bidirectional promoters**. The initial stage identifies orthologous regions between humans and other species. This stage is further refined by defining whether these regions align to non-gapped regions (as nearly perfect matches) or as chained alignments with gaps. After narrowing the region of orthology, genes with orthology can be identified and bidirectional promoters examined. Genes are placed into three categories: Type I genes have orthologous bidirectional promoters surrounded by orthologous pairs of genes. Type II genes have one of two of the orthologous genes in the gene pair and therefore no bidirectional promoter based on the ENSEMBL gene annotations. Type III genes have no orthologs in the ENSEMBL annotations.

Human protein-coding genes regulated by bidirectional promoters were placed into 821 pairs and mapped to other species using conserved synteny information applied through the approach outlined in Figure 1. Three types of outcomes were detected in the species being compared to human, including (I) the orthologous bidirectional gene pair was present in the second species (II) only one member of the gene pair was present in the second species and (III) no evidence existed for a bidirectional promoter in the second species. Comparisons were between human, chimp, rhesus, dog, mouse, chicken, *Fugu*, and zebrafish.

**Figure 2:**
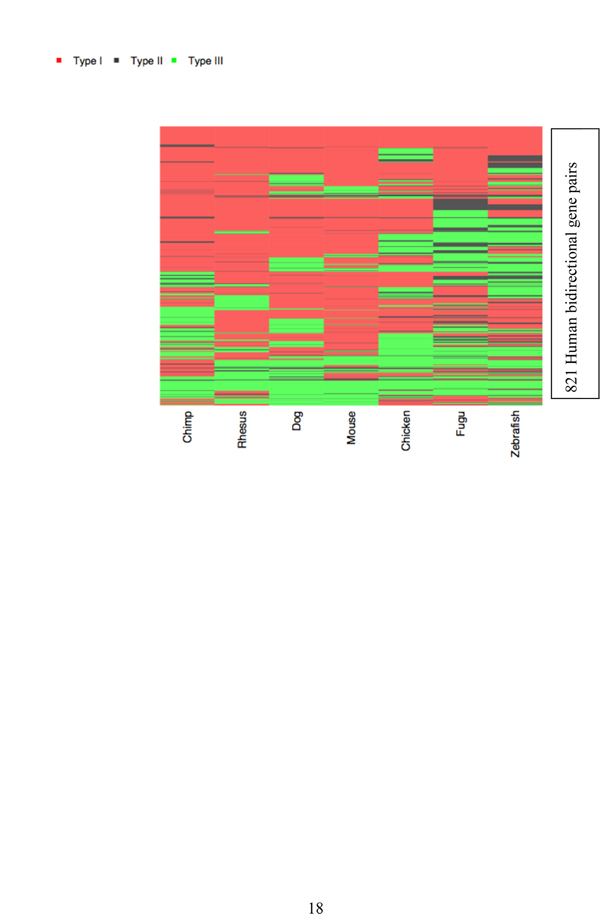
**Mapping the evolutionary history of bidirectional promoters**. Human bidirectional promoters were mapped by their surrounding orthologous genes. Examples marked by a red bar correspond to orthologous bidirectional promoters, where both human genes are present in another species (Type I regions). Regions containing only one ortholog in the pair and no bidirectional promoter (Type II) are marked in black. Type III regions have no evidence of either orthologous gene identified in the human annotations.

Figure 2 illustrates the evolutionary history of bidirectional promoters in vertebrates. For instance, 60 pairs of human genes showed orthologs in all seven species. Another set of genes had orthologs in mammals and fish, but not birds, suggesting evidence for lineage specific loss. Other examples had gene pairs lost only in *Fugu *or zebrafish, suggesting missing annotations in one fish or another. One other set of genes was absent from dog annotations, but present in primates and mouse, suggesting that these genes were missing from the dog genome annotations.

Other sets of bidirectional promoters showed a lineage-specific history. For instance, a large group of mammal-specific genes was not present in chickens or fish. A smaller group was only present in primates. In contrast, genes that were present in all species except chimp were likely to be missing from chimp due to assembly problems. Nearly twenty pairs of genes were found only in the human genome.

### Intergenic distance at bidirectional promoters

**Figure 3:**
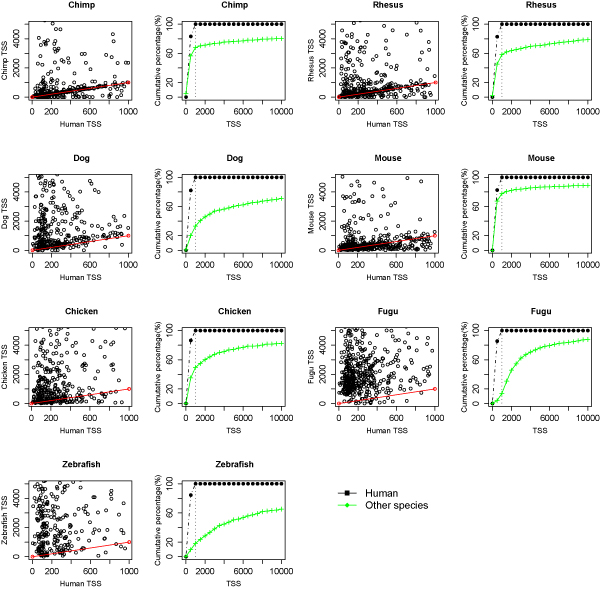
**Distance mapping between orthologous bidirectional promoters**. Each species is compared to the human dataset in two graphs. The left graph plots the distance between transcription start sites for human and the second species at orthologous bidirectional promoters. Human TSSs are limited to 1000 bp intergenic distances. The red lines in the left plots represent bidirectional gene pairs with the same distance between the TSSs in both species. The right graph shows the cumulative percent of the human bidirectional promoters mapped in the second species, allowing a long distance between the TSSs in the second species.

The distance between TSSs at bidirectional promoters was mapped for human and other vertebrate species. Each species is shown in two graphs. One graph depicts the raw distance measurements between the TSS in human and the second species (Figure 3). The distance measurements are graphed with human on the x-axis and the second species on the y-axis. The scatter plots indicate the size of the datasets and the correlation of the bidirectional promoter lengths at orthologous gene pairs of eight species. The red line shows the position of a linear relationship (x = y), where the distances between the TSSs are the same for the two species.

The second graph shows the cumulative percentage of bidirectional promoters mapped in human and a second species, where the human dataset is limited to a 1000 bp distance. The most complete annotations were found in the human-mouse comparison. This result is illustrated by the similar curves for the cumulative percentage of orthologous bidirectional promoters in mouse that fall within 1000 bp. Up to 80% of all human bidirectional promoters were identified in mouse at this similar distance. In comparison, 75% of the human promoters were present in chimp within 1000 bp. The high levels of orthology found in mouse and chimp suggest that the 1000 bp distance will capture similar gene sets in other species. Thus we predict that the gene annotations of chimp, rhesus and dog will improve to represent a minimum of 80% of the bidirectional promoters the human genome.

### Evolutionary comparison of head-to-head and tail-to-tail gene pairs

**Figure 4:**
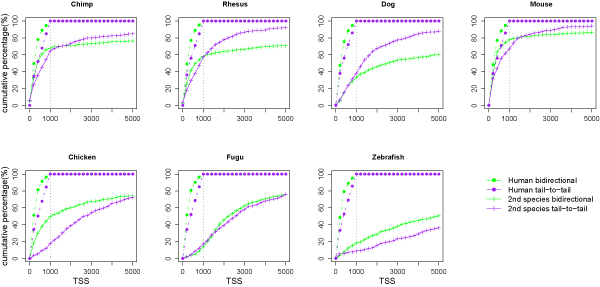
**Comparison of head-to-head and tail-to-tail gene pairs identified at orthologous positions**. Bidirectional promoter data are graphed in green, with dots representing human and plus signs representing the other species. Tail-to-tail gene pairs are represented by purple markers in each species. All data are graphed as the cumulative percentage of the total number of orthologous regions mapped in the human genome.

The percentage of human bidirectional promoters detected at distances up to 1000 bp was compared to the cumulative percentage detected in other species (Figure 4). Evidence of selective pressure was determined from the retention of human tail-to-tail genes, spaced within the 1000 bp limit, in other species. Pairs of genes representing bidirectional promoters are shown in green and tail-to-tail genes in purple. The same color scheme was used for the second species, except that different symbols were used. Although the total percentage of genes mapped in the second species was less than 100% for chimp, rhesus, and dog, the head-to-head and tail-to-tail gene sets had equivalent amounts at the 1000 bp distance. In these datasets the tail-to-tail genes plateau at a longer intergenic distance than the head-to-head genes. Thus a larger distance between the orthologous genes has been tolerated without deleterious effects.

For chicken datasets the head-to-head gene sets were found more frequently than the tail-to-tail sets at 1000 bp, indicating that tail-to-tail arrangements of genes had been allowed to change in both distance and arrangement more often than head-to-head genes. These results indicate that selective pressure acts more strongly over evolutionary time to keep head-to-head genes together at the 1000 bp distance compared to tail-to-tail genes.

The data from the fish genomes indicated that very long distances were necessary to capture a majority of the human gene pairs. Given the compact nature of the fish genomes, it is unlikely that many of these long distance associations are biologically relevant. However, the preservation of tightly associated genes indicated the presence of important regulatory or functional roles that cannot be disturbed.

### Gene ontology associated with bidirectional promoter regulation

Functions associated with orthologous genes regulated by bidirectional promoters were examined for those conserved in all seven species, or in the four mammals. Sixty pairs of genes were conserved across all seven species. These genes were examined for functional classifications. Four groups emerged: intracellular membrane bound organelle, macromolecule metabolism, chaperone, and mitochondrion. The p-values on these groups ranged from 10E-3 to 10E-1, and remained statistically significant following Benjamini correction for false discovery rate (i.e. ~2.7E-1).

Genes that were conserved across the four mammals had a much larger range of functional activities. Of 342 pairs of genes, catalytic activity emerged as the most significant enrichment in any functional class (6.1E-4 after Benjamini correction). Thus bidirectional promoters are regulating many enzymes in mammalian genomes. In total, 58 functional classes were significantly enriched in this dataset compared to a random collection of genes. These data indicate that the regulatory domain of bidirectional promoters has expanded to encompass a much larger set of gene functions in mammals.

### Training ESPERR to discriminate bidirectional promoters

Our previous work indicated that sequence-based characteristics were different in bidirectional promoters and non-bidirectional promoters [7]. However the size of the datasets was quite disparate (1,005 bidirectional, 17,613 non-bidirectional). Therefore for training ESPERR [8] we sampled equal size subsets of 800 elements from each class (keeping the remaining elements in each class as test sets for verification). For each training interval we then extracted genomic alignments of six species (human, chimpanzee, macaque, mouse, rat, and dog) from the 17 species alignments available in the UCSC Genome Browser. Regions of the training data overlapping coding exons (from UCSC Known Genes) were masked out. We first performed an unsupervised encoding selection (the first stage of the ESPERR procedure) to create an encoding in 10 symbols. Leave-one-out cross validation on the training data using this encoding yielded a success rate of 76%. On the bidirectional test set, the model trained using this mapping correctly classified 404 elements (89%) and incorrectly classified 50 (17 elements were not included due to insufficient alignment). On the non-bidirectional test set it successfully classified 11,150 elements (70%), and incorrectly classified 4,845 (687 elements not included). Next, we performed the full ESPERR procedure, using the first stage reduction to produce an encoding of size 75, which was then refined with the heuristic search yielding an encoding of size 10. The resulting encoding gave a modest improvement in cross-validation, with a success rate of 82%. However, on the bidirectional test set, the model using this encoding classified 405 elements (89%) and incorrectly classified 49 (17 elements were again not included due to insufficient alignment). On the non-bidirectional test set it successfully classified 10,900 elements (68%), and incorrectly classified 5,095 (687 elements not included). Thus, using the ESPERR heuristic search gives no improvement for classifying this dataset. It is noteworthy that these classification rates, though modest, indicate that there are sequence and evolutionary patterns that can be captured to characterize bidirectional promoters. Particularly interesting is the substantially greater generalization rate for the bidirectional test set, suggesting that there are more characteristic signals for these elements that can be captured. This is consistent with the result of the ESPERR heuristic search – optimizing the encoding using the training data gives a slight improvement in recognizing the bidirectional test elements, but at the cost of poorer performance on the non-bidirectional test set.

## Conclusion

Our study of bidirectional promoters across orthologous regions of eight species provides a foundation for optimized annotations of these regulatory regions in higher vertebrates, including chimp, rhesus and dog. Furthermore the functional analyses of genes regulated by these promoters show that a small subset of specialized functions in chickens and fish was expanded in mammals to include a wide breadth of activities. A common regulatory mechanism is likely to exist that coordinately regulates genes in these functional pathways. We continue to investigate the features associated with bidirectional promoters using a classification procedure containing supervised and unsupervised techniques. The results are promising in that they indicate that bidirectional promoters have features consistent with learnable patterns.

## Methods

### Assigning orthologous regions

A multi-stage approach to mapping orthology at bidirectional promoters was developed. Because orthology assignments are strongest in coding regions, we mapped single human genes from head-to-head gene pairs to a second species. To identify orthologous DNA we used "chains and nets" data from the UCSC Genome Browser mysql tables. *Chains *in the Genome Browser represent sequences of gapless aligned blocks. *Nets *provide a hierarchical ordering of those chains. Level 1 chains contain the longest, best scoring sequence chains that span any selected region. Gaps in the level 1 chains are recorded in the level 2 chains (of the Browser mysql tables). This ordering process is repeated until all aligned sequences are assigned to a homologous human region. Odd number levels represent aligned regions and even number levels correspond to gapped regions separating the best scoring chained alignments.

We used orthologous regions present in only level 1 and excluded any other levels, which contain both paralogous (duplicated during evolution) and orthologous sequences. Level 1 alignments contain extremely long stretches of genes in conserved synteny (i.e. same gene identity and location) between species. The regions of conserved sequence forming these alignment blocks are separated by gaps that provide spacers between them. Frequently the aligned regions correspond to exons and gapped regions correspond to introns and intergenic regions. Given a human gene, our approach examined whether it fell within an orthologous region defined by level 1 alignment data without knowledge of the exact position within an alignment or on which side of a gap. In a subsequent step, we intersected the positions of gaps and exons of each gene to (1) identify the orthologous gene in the second species and (2) to determine how well the exons align between species.

### Mapping orthologous genes

After determining the orthology assignments using the UCSC alignment data, we used the Ensembl annotations [9] to search the identity of genes within each corresponding alignment. The appearance of several genes in the same region was handled by choosing the candidate with the closest transcription start site to its neighboring gene. This technique identified the most likely gene pair for regulation by a bidirectional promoter. Orthology assignments were checked for each human gene individually, and subsequently checked to see if the pairs from human also formed pairs in the other species. Once orthologous genes were identified for both human genes forming a pair, the orthology assignments were checked in the reverse direction from the other species to human.

Eight species were used for orthology mapping in this analysis including human, chimp, rhesus, dog, mouse, chicken, *Fugu *and zebrafish. Each species has data in the UCSC *chains and nets *data under the hg17 genome assembly, and Ensembl gene annotations. By examining the presence of pairs of orthologous genes in 5 mammals and 3 additional vertebrates, we were able to identify the corresponding orthologous bidirectional promoter regions. This information enabled our investigation of the role of evolution in shaping bidirectional promoters and the types of genes they regulate. Table 1 shows the number of Ensembl gene annotations for each species for our analysis. Table 2 shows the number of orthologous genes that correspond to human genes regulated by bidirectional promoters.

**Table 1:** Count of Ensembl gene annotations used to find orthologous bidirectional promoters

**Mammals**	**Ensembl gene**	**Vertebrates**	**Ensembl gene**
Chimp (panTro1)	26,763	Chicken (GalGal2)	18,979
Rhesus (RheMac2)	23,378	Fugu (Fr1)	34,186
Dog (CanFam2)	19,213	Zebrafish (DanRer3)	22,500
Mouse (MM7)	25,852		

**Table 2:** Orthology mapping for 1642 human genes regulated by bidirectional promoters

**Mammals**	**Number of Orthologous Genes**	**Vertebrates**	**Number of Orthologous Genes**
Chimp (panTro1)	1050	Chicken (GalGal2)	848
Rhesus (RheMac2)	1210	Fugu (Fr1)	703
Dog (CanFam2)	1013	Zebrafish (DanRer3)	631
Mouse (MM7)	1232		

### Dealing with special cases

Some orthologous regions aligned perfectly over very long distances in the second species without any gaps. This situation occurred most frequently in close evolutionary comparisons, such as human to chimp or rhesus. The perfect alignments complicated our mapping of individual gene orthology, because there were no breaks to separate genes within the chained alignment region. To circumvent this problem, we used the Liftover tool available at the UCSC genome Browser. The Liftover software converted the genomic coordinates from human to the second species using the genome alignment information at the nucleotide level. This approach converted perfectly aligned genes between human (hg17) and chimp, rhesus, dog, mouse, chicken or zebrafish. Although Liftover data is not available for *Fugu*, it appears that only 11 such genes align perfectly to *Fugu*, which is a small enough number to handle manually.

Although a singular approach using the Liftover tool could replace the procedure of using chains and nets to more precisely identify each orthologous coordinate, we chose to use it as a second phase in the mapping process. In this way more information was retained from chains and nets regarding how well each exon aligned and how well all genes aligned when gapped regions were present. The overall procedure appears in Figure 1.

## Competing interests

The authors declare that they have no competing interests.

## Authors' contributions

LE conceived of the study. MQY implemented the software and performed most of the analyses. JT conducted the analysis using the ESPERR classification. All authors contributed to writing the manuscript.
